# Intermediate-High Risk Pulmonary Embolism: The Use of Riociguat and Inferior Vena Cava Filter in a Situation of Recurrent Embolism following Insufficient Anticoagulation and Fibrinolytic Therapy

**DOI:** 10.1155/2020/4219616

**Published:** 2020-10-15

**Authors:** Anh Khoi Vo, Håkon Reikvam, Helga Midtbø, Jan Ludvig Wirsching, Øyvind Bruserud, Øystein Wendelbo

**Affiliations:** ^1^Department of Medicine, Haukeland University Hospital, Bergen, Norway; ^2^Department of Clinical Science, University of Bergen, Bergen, Norway; ^3^Department of Heart Disease, Haukeland University Hospital, Bergen, Norway; ^4^Department of Radiology, Haukeland University Hospital, Bergen, Norway; ^5^Department of Anaesthesiology and Intensive Care, Haukeland University Hospital, Bergen, Norway; ^6^VID Specialized University, Faculty of Health, Bergen, Norway

## Abstract

Pulmonary embolism (PE) is associated with serious morbidity and mortality. In this case report, we describe a hemodynamically stable patient with submassive PE and a large thrombus in the inferior vena cava (IVC) protruding into the right atrium (RA), complicated by severe respiratory failure, elevated troponin T (TnT), and right ventricular (RV) dysfunction. The patient was stratified as intermediate-high risk of early death. Important issues regarding the initial choice of anticoagulation, rescue thrombolytic therapy, and benefits of adding riociguat to stimulate the nitric oxide-soluble guanylate cyclase-cyclic guanosine monophosphate (NO-sGC-cGMP) pathway to improve the RV function are discussed. Finally, we address appropriate timing and the use of IVC filter in a situation of recurrent PE following anticoagulation and fibrinolytic therapy.

## 1. Introduction

Venous thromboembolism (VTE) is the third most frequent acute cardiovascular syndrome and the third leading cause of cardiovascular mortality in Western countries [1, 2]. The diagnostic workup usually includes use of Wells score assessment, D-dimer testing, echocardiography (ECHO), and computed tomography pulmonary angiogram (CTPA) [3]. Algorithms for management strategy by the European Society of Cardiology (ESC) involve the assessment of PE severity based on hemodynamic instability and risk of early death [3].

We present a patient with PE and severe respiratory failure. We address appropriate use of anticoagulation, fibrinolytic therapy, and IVC filter and the addition of riociguat, emphasizing the importance of the pathophysiological approach in the treatment of PE.

## 2. Case Presentation

A 49-year-old woman had experienced progressive shortness of breath over a period of two months. She presented to the emergency department (ED) due to acute worsening for the last three days prior to admission. The medical record was deficient; she had undergone nephrectomy as an infant. As adult, she had been diagnosed with hypertension, hypercholesterolemia, absence epilepsy, anxiety, and depression with psychotic symptoms. She also had a previous history of PE nine years prior to this current hospitalization, successfully treated with warfarin for seven months. Laboratory testing for thrombophilia nine years prior was negative. She was a nonsmoker and did not use alcohol, recreational drugs, or herbal medications. Drug prescriptions at the time of admission included simvastatin, losartan, lamotrigin, aripiprazol, escitalopram, oral iron, and vitamin B-supplements. Her body mass index was 46.1 kg/m^2^. Baseline data at presentation in the ED at day one are presented in Table 1.

The electrocardiogram showed sinus tachycardia 130 beats/min, right axis deviation, and T-inversions in the inferior and precordial leads. Bedside ECHO showed signs of severe RV pressure overload with dilatation of the RV and flattened intraventricular septum. The left ventricle was compressed by the RV but had otherwise normal function. CTPA showed extensive, central embolism affecting all pulmonary lobes. A careful examination of the CTPA revealed no thrombus in IVC.

Parenteral anticoagulation therapy with low-molecular-weight heparin (LMWH) was prescribed: 20000 international units of dalteparin daily, immediately following transfer to the Intensive Care Unit (ICU). Blood pressure (BP) fluctuated, but systolic pressure remained > 90 mmHg following intravenous fluid administration, and the lactate values were nearly normalized shortly after.

**Figure 1:**
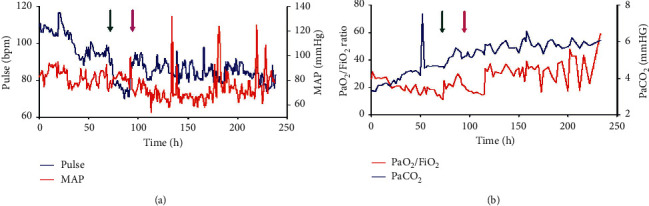
Hemodynamic parameters (MAP and pulse) and respiratory profiles (PaO_2_/FiO_2_ ratio and PaCO_2_) are demonstrated every hour during the 11 days in the ICU for the 49-year-old woman with massive pulmonary embolism. Green arrow indicates the time of systemic thrombolysis. Purple arrow indicates the time of IVC filter implantation. Abbreviations: MAP: middle arterial pressure, PaO_2_/FiO_2_ ratio: arterial oxygen partial pressure to fractional inspired oxygen, PaO_2_: partial pressure of oxygen, PaCO_2_: partial pressure of arterial carbon dioxide, FiO_2_: fraction of inspired oxygen, IVC: inferior vena cava.

**Figure 2:**
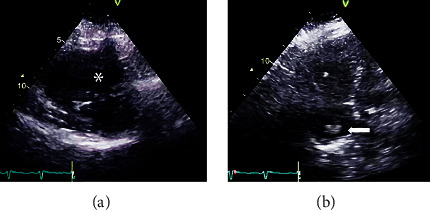
Echocardiography before thrombolysis showed severe right ventricular dysfunction with flattened intraventricular septum (^*∗*^) in parasternal short-axis view (a) and thrombus (arrow) in the right atrium in apical four-chamber view (b).

Hemodynamic parameters and respiratory profiles are presented in Figure 1. On days two and three after admittance, the PaO_2_/FiO_2_ ratio gradually decreased to the lowest value 14.3 (Figure 1). At day three, a mobile structure measuring 1.5 cm × 1.8 cm representing a thrombus was identified in the RA by ECHO (Figure 2). LMWH was then discontinued, and unfractionated heparin (UFH) was prescribed in case of further deterioration and urgent need for systemic thrombolysis.

Due to fluctuating BP and decreasing oxygen ratio on day four, ongoing pulmonary embolization was found likely. Systemic thrombolysis (100 mg alteplase) was administered intravenously. The patient responded suboptimally with transiently increasing oxygen ratio (highest value 24.7). ECHO performed after thrombolysis on day four could no longer visualize the thrombus in the RA. However, signs of RV dysfunction persisted. Tricuspid annular plane systolic excursion (TAPSE) had improved marginally from 17 to 19 mm, and the tricuspid valve peak systolic gradient was measured to 49 mmHg. Riociguat 1 g × 3 was prescribed to relieve and further improve RV function.

**Figure 3:**
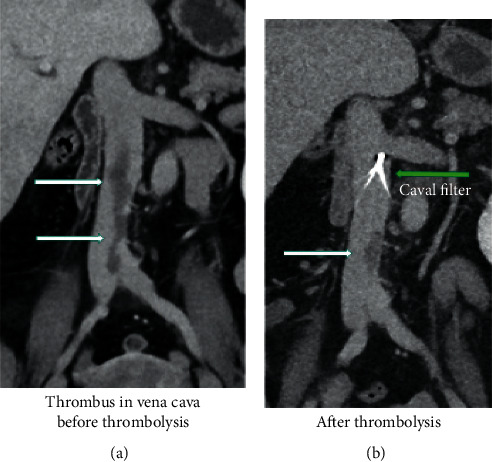
CTPA demonstrating a thrombus of 11 centimetre in the inferior vena cava. (a) The thrombus in the IVC before thrombolysis (white arrows); (b) the residual thrombus (white arrow) following thrombolysis and after insertion of the vena cava filter (green arrow). Abbreviations: CTPA: computed tomography pulmonary angiogram, IVC: inferior vena cava.

Following rescue treatment with systemic thrombolysis, BP continued to fluctuate and the PaO_2_/FiO_2_ ratio remained critically low. A new CTPA showed regression of the extensive central pulmonary embolism, increasing peripheral pulmonary embolism, and a thrombus in the IVC 11 cm (Figure 3).

Due to suboptimal effect of systemic thrombolysis, possible ongoing embolization, and a thrombus of 11 cm in the IVC, we decided to protect her lungs from further embolization by inserting an IVC filter on day five (Figure 3).

Her condition improved significantly the following days (day 7–11), during which the patient received intermittent continuous positive airway pressure (CPAP). The PaO_2_/FiO_2_ ratio continued to improve and CPAP therapy was tapered. When discharged from the hospital, she was started on treatment with warfarin. Riociguat was continued, and UFH was discontinued. ECHO one month after discharge showed a normalized RV but persistent pulmonary hypertension. CTPA two months after discharge from the hospital showed reduction in thrombus size in the IVC now measuring 3.5 cm in length. Overall, the patient was found to be in a good clinical condition.

## 3. Discussion

Appropriate therapy for PE should be based on the patient's risk of early death (in house mortality/death within 30 days), which is identified using a validated risk stratification. Hemodynamically instability defined as a systolic BP < 90 mmHg for a period >15 minutes, hypotension requiring vasopressors, or clear evidence of shock indicate a high risk of early mortality [3, 4]. Further stratification into intermediate and low early mortality risk is based on the presence of RV dysfunction, elevated cardiac TnT levels, and presence of comorbidities and clinical findings that according to Pulmonary Embolism Severity Index (PESI) may negatively affect the prognosis [3, 5].

Our patient presented with shortness of breath, positive D-dimer, elevated TnT levels, signs of RV dysfunction, and very high PESI score (Table 2). Thus, the patient was stratified as intermediate-high risk of early death according to the ESC guidelines.

The urgent need for reperfusion therapy was deemed a possibility due to the gradually decreasing PaO_2_/FiO_2_ ratio during day two and three. Intravenous protamine sulphate can rapidly reverse the anticoagulant effects of UFH, and she was therefore prescribed UFH to allow for rapid systemic thrombolysis. She remained hemodynamically stable during the 11 days in the ICU. However, she developed increasing respiratory failure and there were ECHO findings of a thrombus in the RA. Initial heparinization had proven inefficient and signs of RV dysfunction indicated an imminent deterioration.

The pulmonary embolism thrombolysis (PEITHO) study investigated the effect of thrombolytic therapy on the risk of early death in normotensive patients stratified as intermediate-high risk of early death. Reperfusion therapy was associated with a significant reduction in the risk of hemodynamic collapse but was paralleled by an increased risk of life-threatening bleeding [6]. Primary thrombolysis is therefore not recommended in intermediate-high risk patients without hemodynamic collapse [3]. However, due to increasing respiratory failure, alteplase was administered, which subsequently caused the disappearance of the RA thrombus on ECHO.

The primary cause of death in severe PE is RV failure due to the acute pressure overload [3]. This is due to the combination of mechanical obstruction and pulmonary vasoconstriction [3, 7]. Most treatment options for acute PE focus on the removal of the thrombus; however, pulmonary vasoconstriction, which contributes significantly to RV dysfunction, is often left untreated.

**Figure 4:**
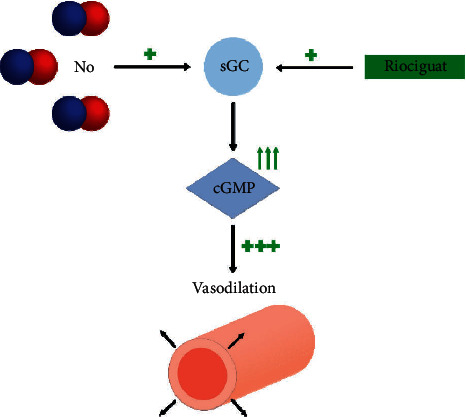
The figure demonstrates a simplified NO-sGC-cGMP pathway. Compromised endogenous NO synthesis and signaling is involved in the pathogenesis of pulmonary hypertension [8, 9]. Both NO and riociguat stimulate sGC which increases the levels of cGMP. This relaxes smooth muscle cells, induces vasodilation, and reduces PVR. Abbreviations: NO: nitric oxide, sGC: soluble guanylate cyclase, cGMP: cyclic guanosine monophosphate, PVR: pulmonary vascular resistance.

The NO-sGC-cGMP pathway is involved in the pathogenesis of pulmonary hypertension [8, 9]. Stimulation of the NO-sGC-cGMP pathway results in increased levels of cyclic guanosine monophoshphate, causing vasorelaxation (Figure 4) [10, 11]. A small number of clinical case reports and experimental studies using animal models show promising results in lowering the pulmonary vascular resistance (PVR) when stimulating the NO-sGC-cGMP pathway [11]. This may in turn reduce the RV strain and dysfunction. Our patient received riociguat, which stimulates the NO-sGC-cGMP pathway by directly stimulating soluble guanylate cyclase and increasing the sensitivity of soluble guanylate cyclase to nitric oxide [8, 12]. This is used in treatment of inoperable chronic thromboembolic pulmonary hypertension (CTEPH) [3, 13], but to our knowledge, there are no clinical reports on the use of riociguat or other sGC-stimulation in acute PE in humans. It is pathophysiologically reasonable that riociguat contributed to recovery of the patient's RV function by reducing PVR and afterload. However, further clinical studies are necessary to elucidate the effects of riociguat in this setting.

After a few days, her respiratory failure worsened dramatically. CTPA follow-up showed regression of the extensive central pulmonary embolism but increasing peripheral pulmonary embolism and an elongated 11 cm thrombus in the IVC. A decision was made to insert an IVC filter. Common indications for IVC filter are VTE and absolute contraindication to anticoagulant treatment, primary prophylaxis in patients with a high risk of VTE, and recurrent PE despite adequate anticoagulation [3, 14]. The aim of IVC filter was to prevent recurrent embolization.

Only two phase III randomized trials have compared anticoagulation with or without vena cava interruption in patients with proximal DVT, with or without associated PE [15–17]. The PREPIC II study showed that in patients with PE and IVC filters in addition to anticoagulation, no reduction in risk of symptomatic recurrent PE was shown at three months [15]. It is, however, likely that the IVC filter prevented new embolic episodes.

## 4. Conclusion

The clinical case emphasizes a stratification-based pathophysiological approach to a guided treatment strategy in accordance with ESC guidelines to avoid fatal outcome. Thrombolytic therapy may be of benefit to patients stratified as intermediate-high risk of early death following a thorough assessment of risk factors including recurrent embolization with increasing respiratory failure, increasing RV dysfunction, and findings of RA thrombus on ECHO. The IVC filter most likely prevented recurrent embolization, which could have been fatal. However, recovery of the RV function together with the improvement of the gas exchange was likely due to the progressive resolution of the embolic obstructions favoured by heparin and perhaps to an additional reduction in RV afterload caused by the stimulation of the NO-sGC-cGMP pathway.

## Figures and Tables

**Table 1:** Clinical parameters and biochemical findings at admission.

*Clinical parameter (unit)*	Value	Reference range

Blood pressure (mmHg)	98/47	
Heart rate (beats per minute)	132	
Respiratory rate (breaths per minute)	40	
Peripheral blood oxygen saturation (%)^*∗*^	90	

*Venous blood tests (unit)*		
Lactate (mmol/L)	5.5	0.9–1.7
Creatinine (*µ*mol)	145	45–90
Sodium (mmol/L)	136	137–145
Potassium (mmol/L)	4.9	3.5–5.0
Urea (mmol/L)	10.9	2.6–6.4
Leucocytes (109/L)	17.5	3.5–11.0
Neutrophils (109/L)	13.4	1.7–8.2
C-reactive protein (mg/L)	198	<5
Procalcitonin (*µ*g/L)	0.25	<0.10
Troponin T (ng/L)	213	≤14
NT-proBNP (ng/L)	5644	<169
D-dimer (mg/L FEU)	>4.00	<0.50

*Arterial blood gas^*∗∗*^ (unit)*		
Arterial partial pressure of oxygen (kPa)	9.3	>10.1
Arterial partial pressure of CO_2_ (kPa)	2.7	4.5–6.1
pH	7.43	7.36–7.44
Base excess	−10.2	−3–+3
Arterial HCO_3_	13	22–26

Notes: ^*∗*^In room air. ^*∗∗*^Test taken with 4 L/minute oxygen supplement on the nasal cannula.

**Table 2:** Original pulmonary embolism severity index [10].

Parameter	No/yes	Patient's score
Age	−/+age	+49
Male sex	0/+10	0
Cancer	0/+30	0
Chronic heart failure	0/+10	0
Chronic pulmonary disease	0/+10	0
Pulse rate ≥ 110 bpm	0/+20	+20
Systolic BP < 100 mmHg	0/+30	+30
Respiratory rate > 30 bpm	0/+20	+20
Temperature < 36°C	0/+20	0
Altered mental status	0/+60	+60
O_2_ saturation < 90%	0/+20	+20
		=199 points

Risk class (points)	Definition (30-day mortality risk (%))	

I (**≤65**)	Very low mortality risk (0–1.6%)	
II (66–85)	Low mortality risk (1.7–3.5%)	
III (86–105)	Moderate mortality risk (3.2–7.1%)	
IV (106–125)	High mortality risk (4.0–11.4%)	
V (>125)	Very high mortality risk (10.0–24.5%)	
